# Strong apoptotic response of testis tumor cells following cisplatin treatment

**DOI:** 10.1007/s11255-023-03825-5

**Published:** 2023-10-27

**Authors:** Beate Köberle, Svetlana Usanova, Andrea Piee-Staffa, Ulrike Heinicke, Philipp Clauss, Anamaria Brozovic, Bernd Kaina

**Affiliations:** 1 Institute of Toxicology, University of Mainz Medical Center, 55131 Mainz, Germany; 2 Department of Anesthesiology, Intensive Care Medicine and Pain Therapy, University Hospital Frankfurt, 60596 Frankfurt Am Main, Germany; 3 Division of Molecular Biology, Ruđer Bošković Institute, 10000 Zagreb, Croatia; 4 Present Address: Department of Food Chemistry and Toxicology, Karlsruhe Institute of Technology, 76131 Karlsruhe, Germany

**Keywords:** Cisplatin, Tumor cell resistance, Apoptotic signaling, Manipulation of apoptotic signaling, Testis tumor cell lines

## Abstract

Most solid metastatic cancers are resistant to chemotherapy. However, metastatic testicular germ cell tumors (TGCT) are cured in over 80% of patients using cisplatin-based combination therapy. Published data suggest that TGCTs are sensitive to cisplatin due to limited DNA repair and presumably also to a propensity to undergo apoptosis. To further investigate this aspect, cisplatin-induced activation of apoptotic pathways was investigated in cisplatin-sensitive testis tumor cells (TTC) and compared to cisplatin-resistant bladder cancer cells. Apoptosis induction was investigated using flow cytometry, caspase activation and PARP-1 cleavage. Immunoblotting and RT-PCR were applied to investigate pro- and anti-apoptotic proteins. Transfections were performed to target p53- and Fas/FasL-mediated apoptotic signaling. Immunoblotting experiments revealed p53 to be induced in TTC, but not bladder cancer cells following cisplatin. Higher levels of pro-apoptotic Bax and Noxa were observed in TTC, anti-apoptotic Bcl-2 was solely expressed in bladder cancer cells. Cisplatin led to translocation of Bax to the mitochondrial membrane in TTC, resulting in cytochrome C release. Cisplatin increased the expression of FasR mRNA and FasL protein in all tumor cell lines. Targeting the apoptotic pathway via siRNA-mediated knockdown of p53 and FAS reduced death receptor-mediated apoptosis and increased cisplatin resistance in TTC, indicating the involvement of FAS-mediated apoptosis in the cisplatin TTC response. In conclusion, both the death receptor and the mitochondrial apoptotic pathway become strongly activated in TTC following cisplatin treatment, explaining, together with attenuated DNA repair, their unique sensitivity toward platinum-based anticancer drugs.

## Introduction

Most solid cancers in adults are resistant to chemotherapeutic treatment once they have spread. In contrast, metastatic testicular germ cell tumors (TGCT) are cured in over 80% of patients using cisplatin [*cis*-diamminedichloro-platinum(II)]-based combination chemotherapy [1–4]. Besides TGCT, cisplatin is used to treat a variety of malignancies, including bladder, ovarian, head and neck, cervical, and lung cancer [1, 5–7], but its clinical efficacy is restricted due to resistance of the tumor cells. Cisplatin resistance appears to be multi-factorial, being associated with reduced drug accumulation, enhanced drug detoxification, modulation of DNA repair mechanisms, and finally alterations in cisplatin-induced signal transduction pathways, which may allow cancer cells to evade cisplatin-induced cell death. It is generally accepted that the antitumor activity of cisplatin is mediated by its interaction with DNA resulting in DNA lesions which may interfere with DNA transcription and replication [8]. Upon recognizing DNA platinum lesions, cells initiate various signaling pathways, eventually leading to cisplatin-induced apoptosis. At least two distinct pathways have been proposed to contribute to cisplatin-induced apoptosis. Apoptotic processes might be triggered through the extrinsic death receptor pathway or via the intrinsic mitochondrial pathway. Cisplatin-induced extrinsic death receptor signaling is mediated through activation of the Fas/FasL system, which is comprised of the membrane surface receptor Fas (CD95) and its ligand FasL (CD179). Binding of FasL to its receptor Fas will activate signaling pathways that lead to induction of apoptosis through activation of a caspase cascade [9, 10]. The intrinsic mitochondrial pathway is executed by various anti-apoptotic or pro-apoptotic members of the Bcl-2 family proteins [11, 12]. The tumor suppressor protein p53 plays a central role in cisplatin-induced DNA damage response, regulating both the extrinsic and the intrinsic DNA damage signaling. Upon treatment with cisplatin, p53 is phosphorylated and undergoes transient stabilization and activation, leading to transcriptional up-regulation of the Fas receptor (FasR) and genes of the mitochondrial pathway, such as Bax, Puma, and Noxa [13–16].

p53 is mutated in approximately 50% of common human cancers, such as cancer of the breast, colon and lung [17, 18]. In contrast, p53 mutations are rare in TGCT [19]. Alterations in p53 were found solely in cisplatin-resistant TGCT [20]. As inactivation or mutation of p53 renders cells more resistant toward cisplatin [21, 22], it was hypothesized that the lack of p53 mutations might be one factor explaining the therapeutic response of TGCT to cisplatin, as much of the sensitivity toward cisplatin observed in TGCT may be due to a propensity to undergo p53-controlled apoptosis in response to DNA damage. This hypothesis is supported by observations that germ cells which are progenitors of TGCTs are particularly susceptible to apoptosis [23].

Cell lines derived from TGCT, which contain non-mutated p53, retain the sensitivity to cisplatin, indicating that cisplatin sensitivity is an inherent property of these cells [24, 25]. This type of tumor cell, therefore, provides a model system with which factors controlling drug sensitivity and resistance in tumors can be studied. Previously, it was observed that TGCT cells are impaired in the repair of cisplatin-induced interstrand crosslinks (ICLs), indicating that persisting cisplatin DNA damage evokes DNA damage signaling resulting in apoptotic cell death and hence cisplatin sensitivity. Cisplatin-resistant bladder cancer cells, on the other hand, were proficient for the repair of cisplatin-induced ICLs [26]. A detailed understanding of how signal transduction upon cisplatin treatment regulates apoptosis might, therefore, help to identify factors involved in damage signaling as possible targets to sensitize drug-resistant tumors.

In the present study, three testis tumor cell lines were used as model systems for cisplatin-sensitive cancer cell lines and compared to three bladder cancer cell lines which have been shown to be drug resistant [25]. Activation of the intrinsic and the extrinsic apoptotic pathway was investigated following cisplatin treatment. Transfection with dominant-negative FADD (DN-FADD) was performed to block the Fas/FasR system as part of the extrinsic pathway. FADD (Fas-associated protein with death domain) has been shown to be a component of the Fas-mediated cell death pathway [27]. FADD connects the Fas/FasL complex to pro-caspase-8 to form the death-inducing signaling complex (DISC) which results in activation of the extrinsic apoptotic cascade [28]. DN-FADD blocks the Fas/FasL system at the beginning of the apoptotic cascade rendering cells insensitive to Fas-mediated apoptosis. p53 shRNA was applied to silence p53 as member of intrinsic apoptotic signaling. Our data indicate that the sensitivity of tumor cells toward cisplatin is related to the p53 status and depends on Fas/FasL and mitochondrial apoptotic signaling pathways. These observations provide a fuller understanding of the involvement of apoptotic processes for cellular resistance/sensitivity toward cisplatin and might open new opportunities for molecular-based cancer therapy by manipulation of DNA damage signaling pathways in cisplatin-resistant cancers.

## Materials and methods

### Cell culture and cisplatin treatment

833 K, SuSa and GCT27 testis tumor cells, MGH-U1, HT1376 and RT112 bladder cancer cells were as described previously [29]**.** All cell lines were grown in RPMI 1640 medium supplemented with 10% heat-inactivated fetal calf serum, L-glutamine (PAA) and 5% antibiotics (penicillin/streptavidin). Cells were cultivated at 37 °C in a humidified atmosphere of 5% CO_2_ in air. New cultures were initiated from frozen vials every 3 month. For cisplatin treatment, cells were incubated for 1 h in medium containing cisplatin (stock concentration 1 mg/ml dissolved in 0.9% NaCl, Pharmacy University of Mainz Medical Center) at 37 °C in a humidified atmosphere.

### Determination of apoptosis

Apoptosis was determined by flow cytometry analysis. 2 × 10^5^ cells were plated in 60 × 15 mm petri dishes containing 5 ml medium. After 24-h cultivation, cells were treated with a range of concentrations of cisplatin for 1 h, washed with PBS and cultivated for 24 h up to 120 h. Cells were harvested, fixed with ice-cold ethanol (70%) and stored for at least 24 h at − 20 °C. Cells were spun down for 15 min at 4 °C, the pellets were resuspended in PBS and incubated with RNase (final concentration 30 µg/ml) for 1 h at room temperature. Cells were stained with propidium iodide (final concentration 17 µg/ml) on ice and in the dark. The sub-G_1_ fraction was determined by flow cytometry using a Becton Dickinson cytometer. Accumulated data were analyzed using WinMDI Software.

### Caspase activity assay

Caspase activity was investigated using the Caspase Colorimetric Assay Kit by Promokine Heidelberg according to the manufacturer’s protocol. Briefly, 2, 5 × 10^5^ cells were seeded in 6 cm dishes, cultivated for 24 h and treated with cisplatin (20 µM) for 1 h, followed by post incubation periods of up to 96 h. Subsequently, cells were lysed and to detect caspase activity substrates IETD-pNA (for caspase-9), LEHD-pNA (for caspase-8), or DEVD-pNA (for caspase-3) were added. All measurements were repeated at least 3 times.

### Transfection

To investigate the influence of the Fas/FasL pathway on cisplatin-induced apoptosis, 2 × 10^5^ 833 K cells were incubated for 24 h with medium containing Effectene Transfection Kit (Qiagen) and 2 µg of vector dominant-negative FADD (DN-FADD) [30] or 2 µg vector pcDNA 3.1 (Invitrogen) containing no insert. After transfection, the cells were washed and treated with cisplatin (20 µM) for 1 h. Apoptosis was determined 72 h post-treatment. The influence of the mitochondrial apoptotic pathway was investigated using a vector containing p53 shRNA (p53 pSuper-RNAi, Oligoengine). To generate stable p53 shRNA sublines, 2 × 10^5^ 833 K cells were transfected with 1 µg of vector pSuper-RNAi. Stably transfected clones were isolated in medium containing 2 µg/ml puromycin and subsequently subcultivated in medium containing 2 µg/ml puromycin. Experiments were performed in medium without puromycin.

### Immunoblotting

Protein extracts were prepared by lysing 5 × 10^6^ cells in 40 µl buffer containing 50 mM Tris–HCl (pH 7.5), 250 mM NaCl, 1 mM EDTA, 0.1% Triton X-100, 1 × protease inhibitor cocktail (Roche). After 30 min incubation on ice, lysates were centrifuged at 13,000 rpm for 20 min at 4 °C and the supernatant was recovered. Protein concentration was determined by the Bradford method using RotiQuant reagent (Roth). For detection of Bax translocation to the mitochondrial membrane, fractionated protein extracts were prepared. Briefly, 5 × 10^6^ cells were lysed in 500 µl ice-cold digitonin lysis buffer (0.2% digitonin, 10 mM PIPES, 300 mM saccharose, 100 mM NaCl, 3 mM MgCl_2_, 5 mM EDTA, 1 × protease inhibitor cocktail) at 4 °C with gentle shaking. After 25 min cells were centrifuged at 2200 rpm for 10 min, the supernatant containing the cytosolic fraction was stored at − 80 °C. To obtain the mitochondrial fraction, 500 µl ice-cold Triton extraction buffer (0.5% Triton X-100, 10 mM PIPES, 300 mM saccharose, 100 mM NaCl, 3 mM MgCl_2_, 5 mM EDTA, 1 × protease inhibitor cocktail) was added to the pellet, followed by incubation at 4 °C with gentle shaking for another 25 min. The solution was centrifuged at 7000 rpm for 10 min, the supernatant containing the mitochondrial fraction was stored at − 80 °C. For immunoblotting, 50 µg of extract protein was separated by electrophoresis on SDS polyacrylamide gels. Proteins were transferred to Whatman nitrocellulose membrane (Roth) overnight at 4 °C in Tris–glycine buffer (25 mM Tris–HCl, 192 mM glycine, 20% methanol). Primary antibodies used were as follows: monoclonal antibodies were used for detection of PARP-1 (BD Biosciences), RPA2 (ThermoScientific), p53 (BP Pharmingen), Noxa (Calbiochem), Tim44 (BD Pharmingen), FasL (BP Pharmingen), polyclonal antibodies used were for detection of Bax (Calbiochem), cytochrome C (Santa Cruz), ERK (Santa Cruz). Membranes were incubated with the primary antibody overnight at 4 °C, followed by incubation for 1 h with either 1/2000 dilution of peroxidase-labeled anti-rabbit IgG or 1/2000 dilution of peroxidase-labeled anti-mouse IgG (DAKO). Proteins were visualized by ECL detection according to the manufacturer’s instructions.

### Preparation of RNA and RT-PCR

PCR was performed as previously described [31]. In brief, total RNA was isolated using the NucleoSpin RNA II Isolation Kit from Machery and Nagel. In a volume of 20 µl, 1 ng RNA was transcribed into cDNA using the Verso^TM^cDNA Kit (Thermo Scientific), an aliquot of 2 µl cDNA was subjected to RT-PCR. Primer sequences used were as follows: *p21*: 5′T-ACATCTTCTGCCTTAGT-3′ and 5′TCTTAGGAACCTCTCATT-3′; *FasR*: 5′-AAGGGATTGGAATTGAGGAAGACTG-3′ and 5′-GTGGAATTGGCAAAAGAAGAAGACA-3′; *GAPDH-:* 5′-GAAGGTGAAGGTCGGAGT-3′ and 5′-GAAGATGGTGATGGGATTTC-3′*; β-actin:* 5′-TCCGCTGCCCTGAGGCACTC-3′ and 5′-GACCCGCCGATCCACACGGA-3′. RT-PCR was performed using specific primers (MWG Biotechnology) and Red-Taq Ready Mix (Sigma Aldrich). The PCR program which has been used was: 2 min at 95 °C followed by 21–38 cycles of 95 °C for 30 s for denaturation, 51–64 °C for 45 s for annealing and 72 °C for 60 s for elongation. 30 µl of PCR product was analyzed on a 1% agarose gel.

### Statistics

For comparing differences between testis versus bladder cancer cell lines, the unpaired *t*-test was performed.

## Results

### Apoptosis in testis and bladder cancer cells following cisplatin treatment

**Fig. 1:**
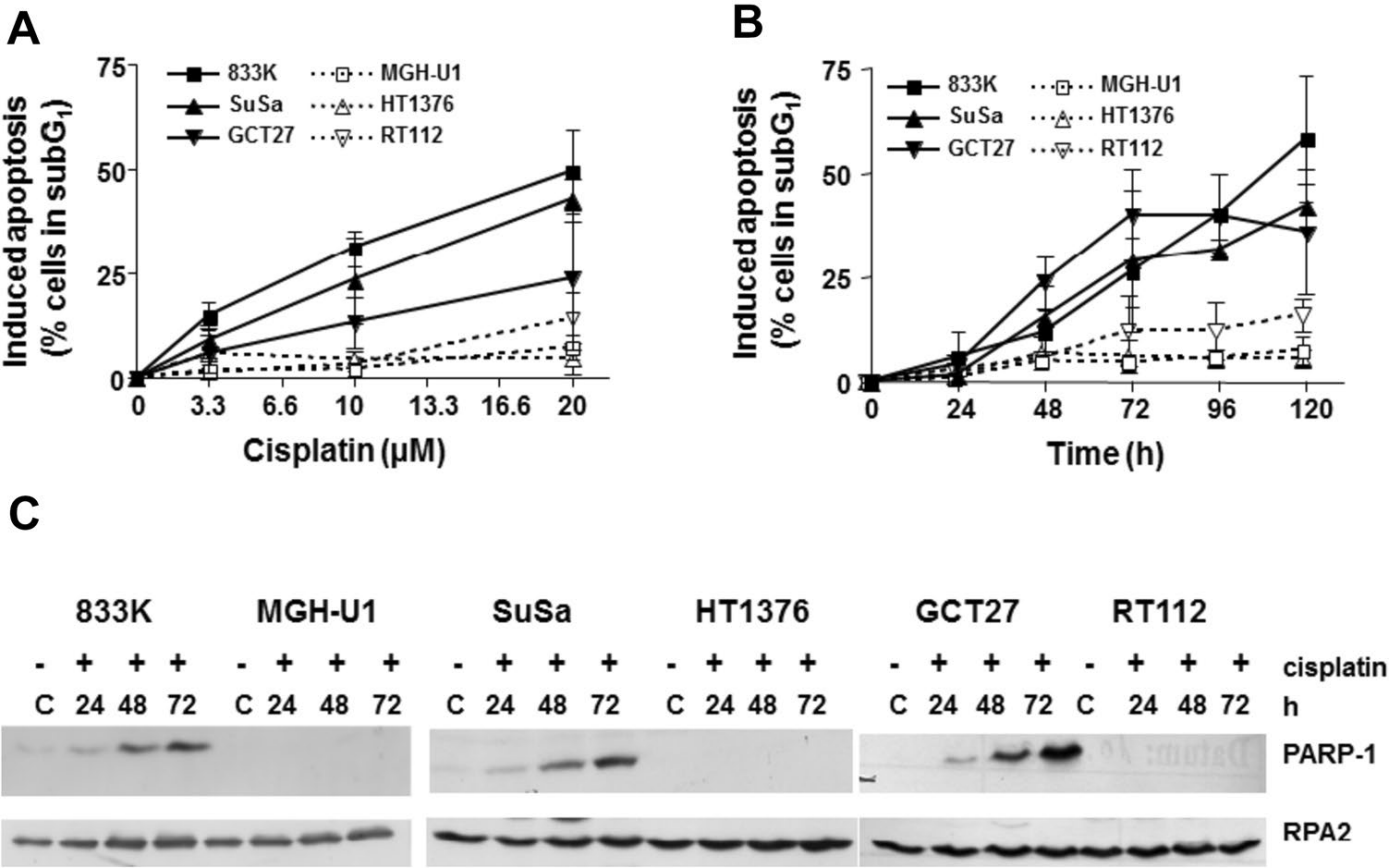
Determination of apoptosis in testis (filled symbols) and bladder (open symbols) cancer cells after treatment with cisplatin. **A** Cells were treated with increasing concentrations of cisplatin for 1 h and post-cultivated for 120 h. Cells were stained with propidium iodide, the sub-G1 fraction was determined using flow cytometry. **B** Cells were treated with cisplatin (20 µM) for 1 h and post-cultivated for different time periods. Cells were stained with propidium iodide, the sub-G1 fraction was determined using flow cytometry. **C** Immunoblot analysis of cleaved PARP-1 in 50 µg protein extract of 833 K, SuSa, GCT27 testis tumor cells and MGH-U1, HT1376, RT112 bladder cancer cells. RPA2 was used as a loading control. Cells were harvested at the indicated time points after treatment with cisplatin (20 µM) for 1 h. C: untreated control

It has been shown that testis tumor cells (TTC) are, on average, threefold more sensitive to cisplatin than bladder cancer cells [25]. The sensitivity of TTC to chemotherapeutic drugs and radiation has been suggested to be largely due to a propensity to undergo apoptosis after DNA damage. Induction of apoptosis by cisplatin was therefore investigated in three TTC lines (8333, SuSa, GCT27) and three bladder cancer cell lines (MGH-U1, HT1376, RT112). The cells were exposed to cisplatin for 1 h and the sub-G1 fraction, which represents apoptotic cells, was measured. A higher dose- and time-dependent induction of apoptosis was observed in testis tumor compared to bladder cancer cells. Cisplatin at concentrations of 10 and 20 µM leads to a significantly higher amount of cells in the sub-G1 fraction in testis versus bladder cancer cells (*P* = 0.0219) (Fig. 1A). Starting 48 h post-treatment, a significantly higher induction of apoptosis was observed in testis versus bladder cancer cells, with *P*-values of *P* = 0.0376 (48 h),* P* = 0.0063 (72 h), *P* = 0.0014 (96 h) and *P* = 0.0082 (120 h) (Fig. 1B). PARP-1 cleavage as a marker of apoptosis induction was clearly more pronounced in the testis tumor cells (Fig. 1C, showing the PARP-1 cleavage fragment), which is in line with the higher apoptotic response in this type of cells following cisplatin treatment. RPA2 was used as a loading control (Fig. 1C). These data extend previous observations of the cisplatin hypersensitivity of testis tumor cells which were obtained using colony formation assays [25] and support the suggestion that the hypersensitivity of testis tumor cells to cisplatin is associated with their enhanced propensity to undergo apoptosis, most likely caused by persisting cisplatin DNA damage due to reduced ERCC1-XPF-mediated ICL repair [26].

### Caspase activation in testis and bladder cancer cell lines

**Fig. 2:**
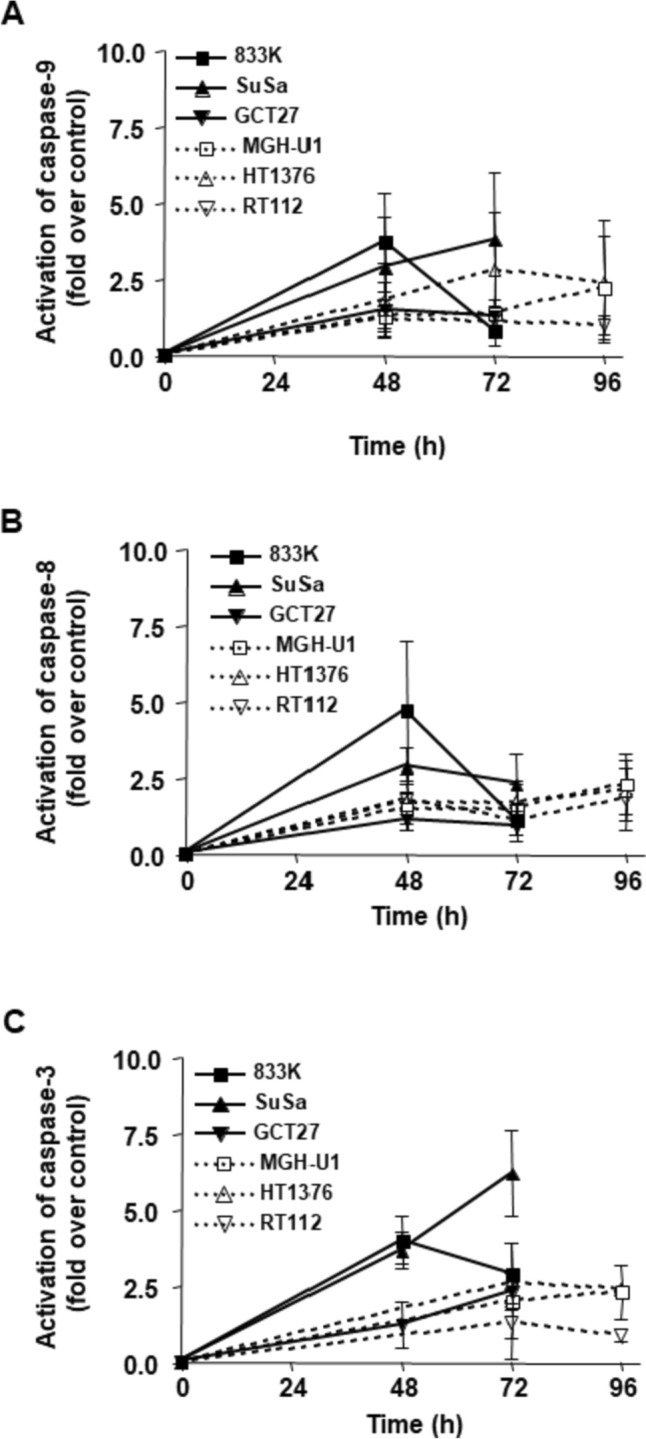
Activation of the initiator caspases-9 and -8 and effector caspase-3 after treatment with cisplatin. Testis tumor cells (filled symbols) and bladder cancer cells (open symbols) were treated with cisplatin (20 µM) for 1 h, activation of **A** caspase-9, **B** caspase-8 and **C** caspase-3 was determined at the indicated time points after treatment. Results are shown as fold activation over untreated control. The line GCT27 is a poor responder. When performing statistical analysis of the two testis lines 833 K and Susa in comparison to the bladder cancer lines, the results are as follows: For caspase-8: the difference between testis and bladder cancer cell lines was not significant (*p* = 0.0544). For caspase-9: the difference was significant (*p* = 0.014). For caspase-3: the difference was significant (*p* = 0.0136)

The mitochondrial and the death receptor pathway have been implicated in mediating apoptosis following DNA damage [32]. To investigate the activation of these pathways, cells were treated with cisplatin (20 µM) for 1 h, and activation of the initiator caspases-8 and -9 as well as the effector caspase-3 was determined at different time points post-treatment (Fig. 2). Caspase-8 is part of the death receptor pathway, while caspase-9 is activated by the mitochondrial pathway, both these caspases lead to activation of caspase-3. Activation of both caspase-9 and caspase-8 was observed in the testis tumor cells 48 h after cisplatin treatment, the bladder cancer cell lines responded later and to a lesser extent (Fig. 2A, B). The difference, however, was considered to be not statistically significant. Caspase-3 was also activated following cisplatin treatment in both testis and bladder cancer cells (Fig. 2C). Altogether, the findings indicate that both pathways are intact and involved in the apoptotic response of testis cancer cells upon cisplatin treatment.

### Mitochondrial pathway in cisplatin-induced apoptosis in testis tumor cells

**Fig. 3:**
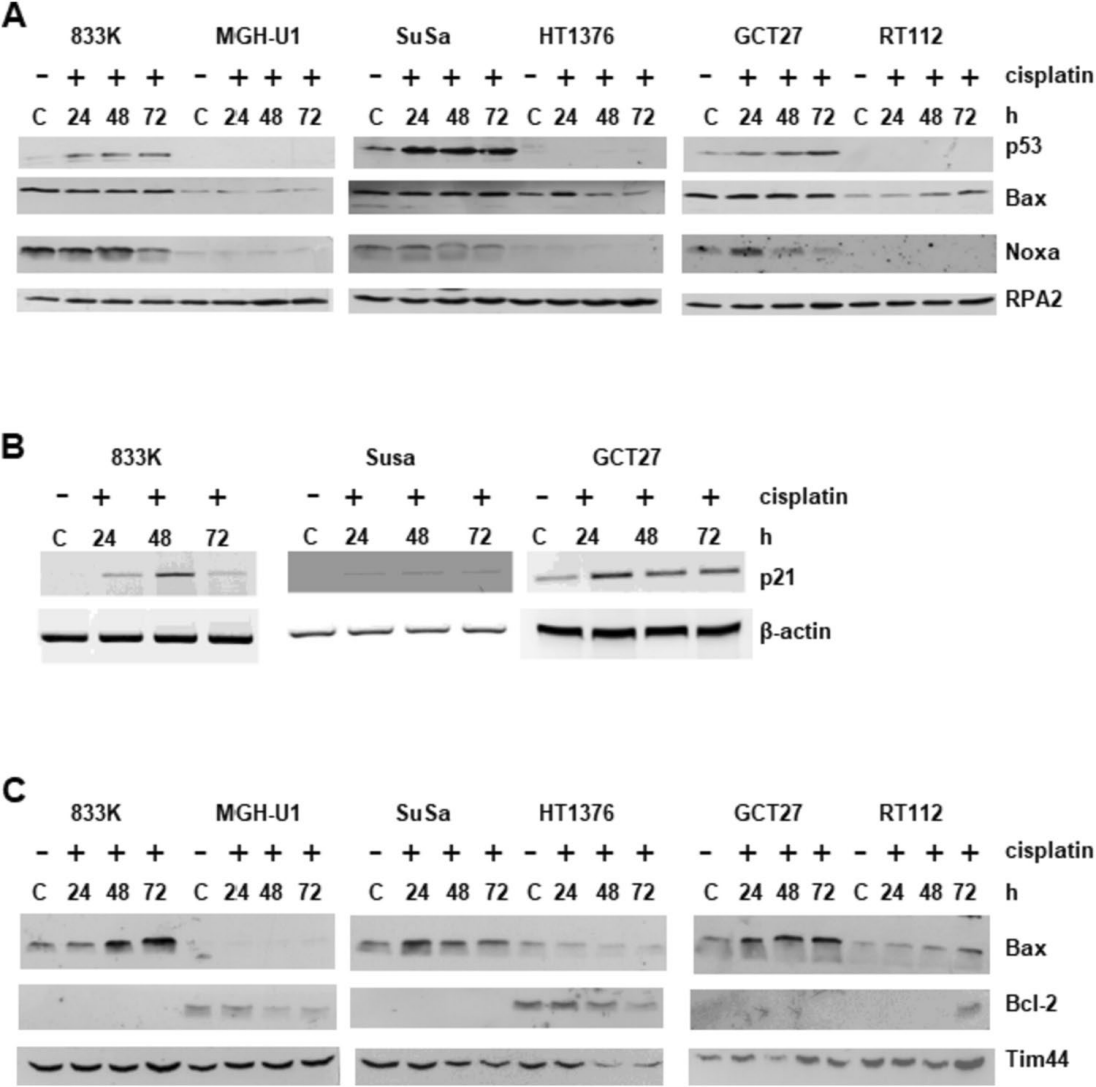
Analysis of pro- and anti-apoptotic proteins in 833 K, SuSa, GCT27 testis tumor cells and MGH-U1, HT1376, RT112 bladder cancer cells. (A) Immunoblot analysis of p53, Bax, Noxa in 50 µg protein extract of cells treated with cisplatin (20 µM) for 1 h. Cells were harvested at the indicated time points after treatment. RPA2 was used as loading control. C: untreated control. (B) mRNA analysis of *p21 gene* in testis tumor cell lines. Cells were harvested at the indicated time points after treatment with cisplatin (20 µM) for 1 h. C: untreated control. *β-actin* was used as a loading control. (C) Immunoblot analysis of Bax and Bcl-2 in 50 µg protein extract (mitochondrial fraction). Cells were harvested at the indicated time points after treatment with cisplatin (20 µM) for 1 h. C: untreated control. Tim44 (translocase of inner mitochondrial membrane 44) was used as a loading control

Next, the activation of the mitochondrial pathway was investigated in TTC and compared to bladder cancer cells. Cells were treated with cisplatin (20 µM) for 1 h and protein levels of p53, Bax, Noxa and Bcl-2 were determined 24, 48 and 72 h post-treatment. p53 levels were increased in response to cisplatin in TTC leading to transcriptional activation of its target gene p21, while no p53 was detectable in the bladder cancer cells (Fig. 3A, B). The cellular levels of the pro-apoptotic proteins Bax and Noxa were not increased in the tumor cells upon cisplatin treatment (Fig. 3A). However, the basal levels of Bax and Noxa were considerably higher in TTC than in the bladder cancer cells. In fractionated cell extracts, Bax was detected in the mitochondrial fraction of protein extracts of testis tumor cells showing an increase following cisplatin treatment, while Bax was barely detectable in the mitochondrial fraction of bladder cancer cells (Fig. 3C). These findings suggest a translocation of Bax protein to the mitochondrial membrane in testis, but not bladder cancer cells following cisplatin treatment. The anti-apoptotic protein Bcl-2, on the other hand, was not detectable in the mitochondrial fraction of TTC, but could be seen in bladder cancer cells (Fig. 3C). Bax dimerization in the mitochondrial membrane leads to permeabilization of the membrane, cytochrome C release and subsequent apoptosis. Altogether, the data support the notion that the mitochondrial pathway is better activated by cisplatin in drug-sensitive testicular cancer cells.

### Fas/FasL pathway in cisplatin-induced apoptosis in testis tumor cells

**Fig. 4:**
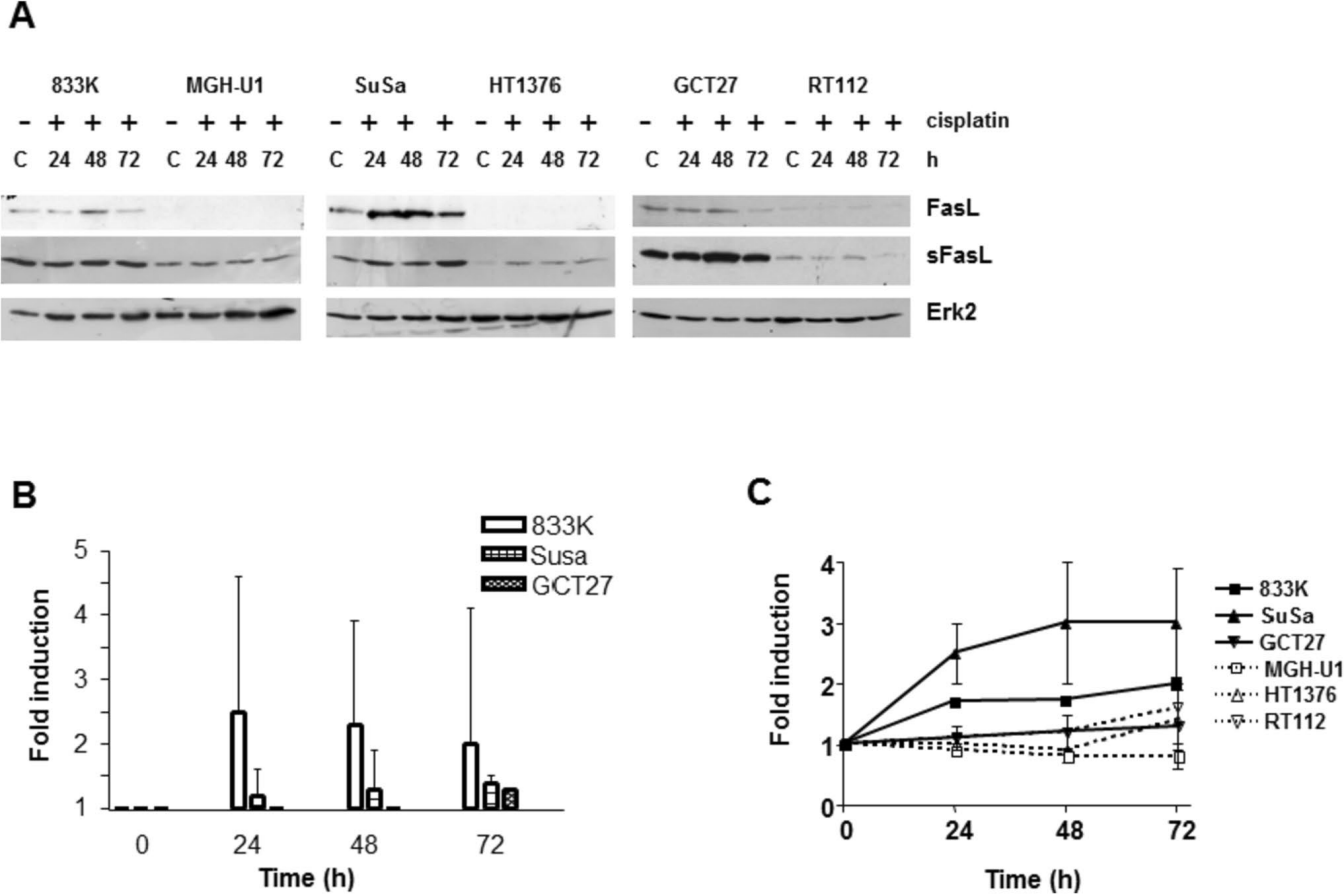
Analysis of Fas/FasL signaling pathway in testis and bladder cancer cells. **A** Immunoblot analysis of FasL and sFasL in 50 µg protein extract of 833 K, GCT27, SuSa testis tumour cells and MGH-U1, HT1376, RT112 bladder cancer cells. Cells were treated with cisplatin (20 µM) for 1 h, cells were harvested at the indicated time points after treatment. ERK2 was used as a loading control. C: untreated control. **B** Quantification of the immunoblots of FasL protein expression in testis tumor cells after cisplatin treatment. Shown are the mean values ± standard deviation of three independent experiments. **C** Quantification of Fas mRNA expression after cisplatin treatment. Cells were treated with cisplatin (20 µM) for 1 h, cells were harvested at the indicated time points after treatment. *GAPDH* was used as a loading control

As caspase-8, which is downstream in the death receptor pathway, is activated upon cisplatin treatment, it was, therefore, investigated whether factors involved in death receptor signaling were regulated in a different way in testicular and bladder cancer cell lines after cisplatin treatment. The death receptor signaling rests on activation of the Fas receptor/ligand system. Treatment with cisplatin increased the level of the membrane-bound Fas ligand (FasL) in the TTC (833 K, SuSa and GCT27), while no FasL could be detected in the bladder cancer cell lines (Fig. 4A, B). The soluble FasL (sFasL) was expressed in TTC, while only a moderate expression was observed in bladder cancer cells. To analyze the expression of the Fas receptor (Fas), quantitative PCR was applied. Quantification of the data revealed that treatment of cells with cisplatin lead to a two- to threefold up-regulation of Fas in 833 K and SuSa cells and a less than twofold induction of Fas in GCT27 testis and RT112 and HT1376 bladder cancer cells, while no up-regulation was observed in the bladder cancer cell line MGH-U1 (Fig. 4C) supporting the notion that the death receptor pathway becomes also activated following cisplatin treatment in drug-sensitive testis tumor cells.

### Modulating apoptotic signaling in testis tumor cells

**Fig. 5:**
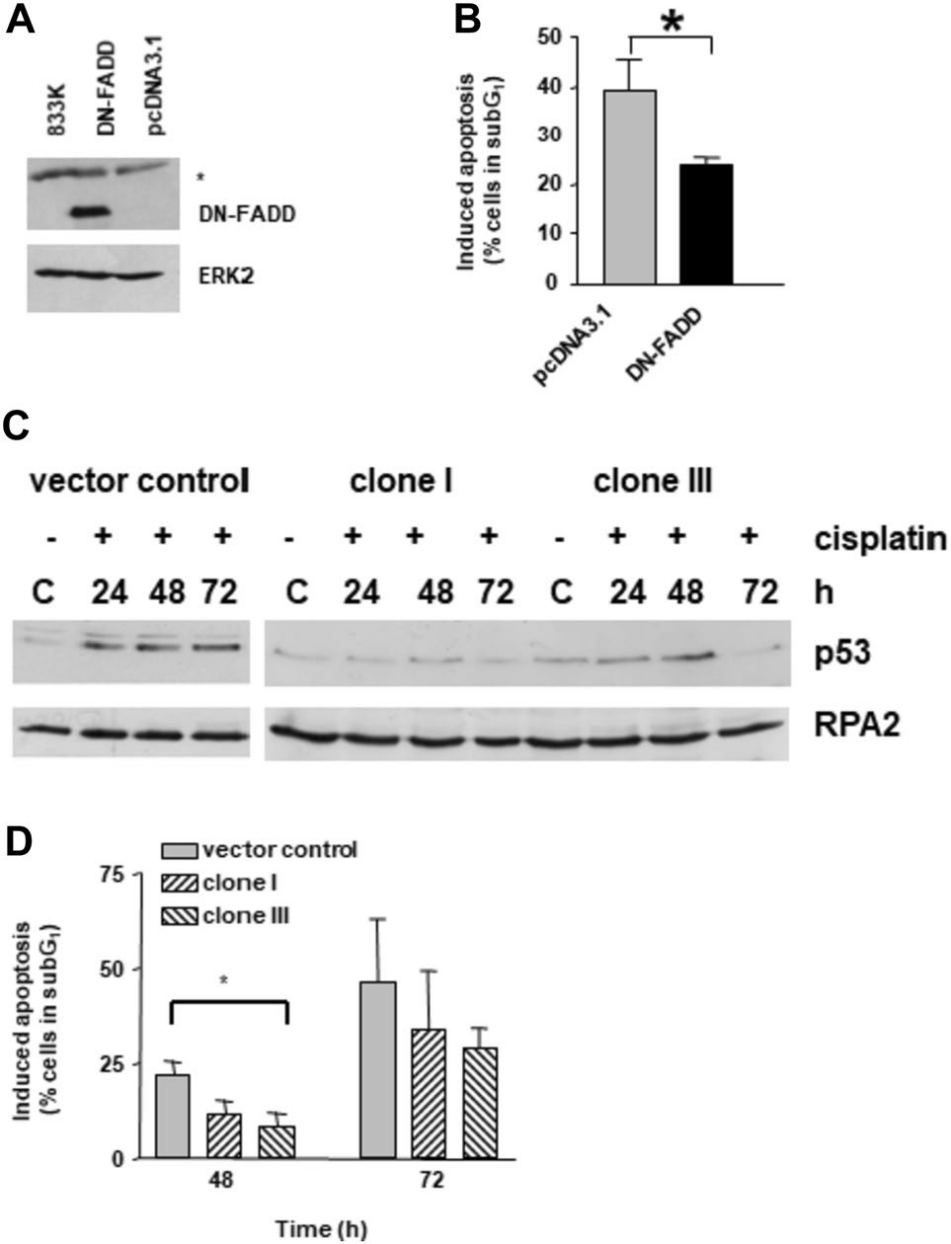
Interfering with apoptotic signaling in 833 K testis tumor cells. **A** Immunoblot analysis of 833 K cells transfected for 24 h with a vector containing dominant-negative FADD (DN-FADD) or an empty vector (pcDNA3.1). 50 µg protein extract were analyzed for the DN-FADD fragment. Asterix: unspecific band. ERK2 was used as a loading control. **B** Determination of apoptosis in 833 K cells transfected for 24 h with empty vector or DN-FADD. Transfected cells were treated with cisplatin (20 µM) for 1 h and post-cultivated for 72 h. Apoptosis was measured by flow cytometry (Sub-G1 content). **C** Immunoblot analysis of p53 in 833 K transfected clones. Cells were transfected with p53 pSuper vector and stably transfected clones were isolated. Cells were treated with cisplatin (20 µM) for 1 h and harvested at the indicated time points. Clone I, III: 833 K cells stably transfected with p53 pSuper-RNAi; vector: 833 K cells stably transfected with pSuper. C: untreated control. RPA2 was used as loading control. **D** Determination of apoptosis in 833 K clones stably transfected with p53 pSuper vector. Clones were treated with cisplatin (20 µM) for 1 h and post-cultivated for 48 h or 72 h. Apoptosis was measured by flow cytometry (Sub-G1 content)

To inhibit death receptor signaling, dominant-negative FADD (DN-FADD) was used. 833 K cells were transiently transfected with an expression vector for DN-FADD, transfected cells were treated with cisplatin and induction of apoptosis was determined. Transfection with DN-FADD (Fig. 5A) decreased the level of induced apoptosis of cisplatin-treated 833 K cells compared to cells transfected with pcDNA3.1 vector control (Fig. 5B), indicating that interfering with the death receptor pathway had a negative effect on apoptosis induction by cisplatin. The decrease in sensitivity was significant, but cells were not completely blocked in apoptosis, most likely because the mitochondrial pathway is involved as well. To investigate the importance of p53 for cisplatin sensitivity in testis tumor cells, p53 was down-regulated in 833 K cells. The cells were transfected with a vector expressing p53 shRNA and stably transfected clones were isolated. p53 shRNA resulted in silencing of p53 protein expression, as treatment with cisplatin did not lead to an increase in p53 levels in the transfected clones (designated as clones I and III) (Fig. 5C). In the vector control clone (transfected with the empty vector), cisplatin leads to an increase in the p53 level comparable to parental 833 K cells (Fig. 5C). To investigate the effect of silencing of p53 on apoptosis induction, cells were treated with cisplatin and induction of apoptosis was determined. We observed a significant difference in apoptosis induction in the vector control clone compared to clones I and III with silenced p53 48 h post-treatment with cisplatin (Fig. 5D). 72 h post-treatment, there was still less apoptosis induction in clones I and III compared to the vector control clone, however, to a lesser extent. Overall, the data support the view that both the Fas and the mitochondria pathway are active in testis tumor cells and contribute to their cisplatin sensitivity.

## Discussion

Previously, we and others showed that TTC are prone to cisplatin treatment [33, 34]. The present study shows that TTC respond to cisplatin treatment by a prompt induction of apoptosis, compared to bladder cancer cells, which are clearly more resistant. Both the death receptor and the mitochondrial apoptotic pathway become activated in TTC upon cisplatin treatment. Previously, it was also shown that TTC are impaired in the repair of ICLs due to reduced levels of ERCC1-XPF resulting in persisting critical DNA damage [3, 26], that trigger DNA damage signaling. Data by Bartkova et al. showed that the downstream ATM and p53-triggered DNA damage response in tissues of testis tumors are intact [35]. This, together with attenuated DNA repair and prompt apoptosis execution, explains the exceptional therapeutic response of this type of tumor, which is a standard example of successful genotoxic chemotherapy. For comparison, we used bladder cancer cells, displaying less vulnerability to apoptosis induction, and a reduced sensitivity toward the drug [25]. It should be mentioned that long-term cisplatin treatment resulted in a robust caspase activation and apoptosis also in bladder cancer cells [36], indicating they are in principle able to execute apoptosis.

Almost all TGCT are characterized by wild-type p53 [19, 37], which is important to note as it was hypothesized that p53-controlled apoptotic signaling is causally involved in the chemosensitivity of TTC. This is supported by observations that TTC from patients who failed cisplatin chemotherapy could be linked to a mutation in p53 [20, 38, 39]. We observed an increase in p53 levels upon cisplatin treatment in our panel of TTC lines, while no p53 was detected in the bladder cancer cells. In addition, silencing of p53 rendered TTC more resistant to cisplatin, supporting the hypothesis that cisplatin sensitivity of TTC is a result of p53-controlled apoptotic signaling. In line with our observations, it was reported that silencing of p53 completely abrogated the cisplatin-induced killing response in NTERA-2D1 testis tumor cells [40]. In addition, a correlation between cisplatin-induced apoptosis and the amount of p53 protein could be demonstrated. A clear association between p53 level and cellular sensitivity was also reported for the UV-mimetic drug 4NQO [41]. These observations indicate that functional p53 is central for apoptosis induction following DNA damage in TTC. We should note that there are also an early conflicting report by Burger et al., who found no association between functional p53 and the propensity to undergo apoptosis after cisplatin treatment in a TTC lines [42]. Furthermore, abrogation of p53 function did not affect cisplatin sensitivity in TTC [43]. In addition, they observed that the expression of the p53 target gene *Bax* was not modulated upon cisplatin treatment, regardless of the p53 status [42]. Based on these findings, the authors concluded that neither p53 nor Bax is involved in cisplatin-induced apoptosis in TTC. Unfortunately, in this study, the Fas pathway was not addressed and the amount of Bax was not compared between cisplatin-sensitive and cisplatin-resistant tumor cells. Thus, TTC lines express high levels of Bax, which is in contrast to bladder cancer cells, and it was hypothesized that high endogenous levels of Bax could contribute to the drug sensitivity of TTC [44]. An important role of p53 for transactivation of Bax following cisplatin has also been reported for other cancer models, such as ovarian cancer [45].

Here, we show that the levels of Bax and Noxa were not upregulated following cisplatin treatment, but the endogenous levels of Bax and Noxa were higher in TTC compared to bladder cancer cells. Also, translocation of Bax to the mitochondrial membrane and cytochrome C release was observed in TTC following cisplatin treatment. Bax exists as a monomer in the cytosol and translocates to the mitochondrial membrane during apoptosis, which is supported by p53 [46]. In a reaction independent of transcription, p53 can directly support translocation and dimerization of Bax, which will lead to permeabilization of the mitochondrial membrane and cytochrome C release [47, 48]. Bax translocation appears to be central to the execution of mitochondrial apoptosis, as inhibitors of Bax translocation to the mitochondria enhanced viability of Chinese hamster ovary cells in response to cisplatin treatment, most likely due to reduced levels of caspase activity [49]. An association between Bax translocation and cisplatin resistance has also been proposed for bladder cancer cells, as inhibition of Bax translocation to the mitochondrial membrane resulted in reduced cell death [50]. For TTC, we conclude that high endogenous levels of Bax favor translocation to the mitochondrial membrane and p53 exerts its pro-apoptotic role by activation, rather than transcription of Bax. It is important to note that high expression of Bax and apoptotic activity was also observed in clinical samples of testis tumor tissues, supporting the notion that Bax affects the drug response in this type of cancer [51].

The *Fas* receptor gene is regulated by p53 [52]. Thus, studies revealed binding of p53 to the *Fas * promoter causing activation of the gene and Fas pathway activation, while mutant p53 failed to induce apoptosis via Fas [53]. We found that apoptosis induced by cisplatin in TTC is supported by Fas, while bladder cells showed only low or lack of Fas induction. The soluble Fas ligand (sFasL) was strongly expressed in TTC and moderately expressed in bladder cancer cells. While FasL is essential for activation of the exogenous apoptosis pathway, sFasL generated through cleavage of FasL by metalloproteinases has reduced apoptotic activity [54]. Silencing of the Fas/FasL-driven death receptor pathway by transfection with dominant-negative FADD attenuated the cytotoxic effect of cisplatin in the 833 K testis tumor cell line, supporting again  that activation of Fas/FasL signaling contributes to cisplatin sensitivity of testis tumor cells. The importance of this pathway for drug sensitivity has also been reported for other tumor lines [55]. Thus, FasL was upregulated in cisplatin-sensitive ovarian carcinoma cells, and the sensitivity toward cisplatin could be reduced by modulating Fas/FasL [56]. For the testis tumor cell line Tera 1, it was shown that p53 upregulated Fas and triggered effectively apoptosis [57]. We also observed up-regulation of FasL, which did not occur in cisplatin-resistant bladder cancer cells. Of note, there are many other examples of Fas independent apoptosis, e.g., in lung cancer cells [58, 59], indicating cell-type specificity.

Various approaches to target the Fas/FasL pathway have been reported [60]. Application of activating Fas antibodies leads to enhanced apoptotic death in vitro [61], however at the expense of severe systemic toxicity in a mouse model [62]. Another mechanism to manipulate the Fas pathway has been reported for TNFα. Treatment with TNFα enhanced cisplatin cytotoxicity in neuroblastoma cells, most likely through the activation of NF-κB which in turn induced transcription of *Fas* and hence apoptotic signaling [63]. Further studies will show whether these strategies can be utilized to increase the effectiveness of cisplatin in TTC therapy.

As outlines above, TGCT is a tumor characterized by frequent non-mutated p53, which is  harnessed for cancer therapy as p53 is a driver of apoptotic signaling. At the same time, the question arises as to the mechanism of carcinogenesis in the presence of wild-type p53. Testicular cancers are malignancies of primordial germ cells arising from the precursor lesion GCNIS (germ cell neoplasia in situ) under the influence of genetic, epigenetic and environmental factors [64]. The question of why p53 is not contributing to the oncogenic process is challenging, but seems to be unresolved.

## Conclusion

Cell lines derived from cisplatin-sensitive testicular cancer, in which DNA repair is attenuated due to downregulation of ERCC1/XPF, undergo highly efficient apoptosis following cisplatin treatment. Both the intrinsic and the extrinsic apoptotic pathway became activated. Blocking the extrinsic Fas system decreased the level of apoptosis, rendering TTC more resistant. Similarly, silencing of p53, which drives the intrinsic apoptotic pathway reduced cisplatin sensitivity. Overall, the data suggest that impaired nucleotide excision repair, active DDR, functional p53 and effective apoptosis execution through the Fas and the mitochondrial pathway explain the exceptional TTC drug sensitivity. Translation of data to cisplatin-resistant cancers might broaden the therapeutic window of this chemotherapeutic drug.
